# Precision management of pollination services to blueberry crops

**DOI:** 10.1038/s41598-021-00068-1

**Published:** 2021-10-14

**Authors:** P. Cavigliasso, P. Negri, M. Viel, M. M. Graziani, C. Challiol, F. Bello, A. Saez

**Affiliations:** 1 Instituto Nacional de Tecnología Agropecuaria (INTA), EEA Concordia - División Frutales, Concordia, Entre Ríos, Argentina; 2 Instituto de Investigaciones en Producción Sanidad y Ambiente (IIPROSAM), CONICET-UNMdP, Centro de Asociación Simple CIC PBA, Buenos Aires, Argentina; 3 Centro de Investigaciones en Abejas Sociales, Facultad de Ciencias Exactas y Naturales, Universidad Nacional de Mar del Plata, Deán Funes, Argentina; 4 Beeflow Inc. - Smart Pollination Services, Los Angeles, CA USA; 5 Facultad de Ciencias de la Alimentación, Universidad Nacional de Entre Ríos (UNER), Concordia, Entre Ríos, Argentina; 6 Grupo de Ecología de la Polinización, INIBIOMA (CONICET-Universidad Nacional del Comahue), San Carlos de Bariloche, Rio Negro, Argentina

**Keywords:** Agroecology, Plant reproduction

## Abstract

While the cultivated area of pollinator-dependent crops is increasing, pollinator availability is decreasing, leading to problems in many agroecosystems. For this reason, pollinator-dependent crop growers often rent beehives to support their pollination requirements to sustain fruit productivity. However, the efficiency of those pollination systems has not been extensively studied. Here, we compared the effect of “precision” pollination (i.e., application of pesticides coordinated with growers, audit of hives, dietary supplementation and individual distribution of hives) with conventional practices (i.e., pesticides applications without coordination with growers and no audit of hives, low maintenance of hives and hives distributed in large groups) on the mean level of pollination and fruit production and quality in blueberry crops. In nine blueberry fields, we measured bee visitation rate to flowers, fruit set, fruit firmness and fruit weight. On average, precision-pollinated plots had 70% more bee visits to flowers and produced 13% more fruits that were 12% heavier and 12% firmer than those obtained through conventional practices. These results showed that pollination efficiency could be improved if key management related to bee strength, distribution and health care are taken into account. Due to these results, we encourage growers and beekeepers to include precision pollination practices to both increase the productivity of blueberry fields and the wellbeing of honey bees within agroecosystems.

## Introduction

There is growing evidence that the ecosystem services provided by the entomofauna are at risk^1^, despite the great economic and environmental benefits they bring to humans^2^. Based on the work carried out by Klein et al. (2007)^3^, detailed studies in agroecosystems highlighting the relevance of the ecosystem service of crop pollination for fruit productivity and quality are becoming more frequent^4–6^.

In areas where intensive agriculture is practiced, the abundance of populations of *Apis mellifera* are highly threatened, observing a decrease of this species on a global scale^7,8^. These trends have increased concern at political and institutional levels (e.g., Food and Agriculture Organization, Intergovernmental scientific-normative Platform on Biodiversity and Ecosystem Services) and have increased the urgency to improve our understanding of how pollinating insects behave within agroecosystems^9^ and ways to mitigate the loss of pollination services^10–12^. Unfortunately, studies dealing with this topic are scarce, although they are priorities for the management and optimization of pollination in crops of high economic importance^13,14^.

The first measure implemented to fight pollination deficiencies in high-dependency productive systems^15^ was the supplementation of managed hives of *A. mellifera*. It has been demonstrated that the introduction of beehives in productive farms significantly increases fruit production^16–19^. This alternative management is widespread in most crops of middle to high levels of pollinator dependence, where the need for a vector for pollen transfer is essential. Even so, this practice does not always yield positive results for both interacting partners (i.e., growers and beekeepers). Growers may not receive efficient pollination services if hives are low quality (i.e., have low number of frames covered with bees, or bees with high levels of pests) and/or beehives are placed far from the field to pollinate^20^. Beekeepers, on the other hand, may lose many hives if pesticides are applied during flowering (i.e., colony loss by intoxication)^21^ and/or if flower resources within or surrounding the farm are not sufficient in terms of abundance or quality to sustain their well-being (i.e., colony loss by starvation)^22^. Proper management of the pollination service entails different practices, which start before the floral bloom and before the introduction of the beehives to the fields and continue throughout the period of flowering, including cultural practices (defined as techniques or management options that can be implemented to achieve an objective, in this case the pollination efficiency) in both the colonies and the crop^22–24^.

In the northeast of Argentina, between the provinces of Corrientes and Entre Rios, there are 1050 ha of blueberry crops *Vaccinium corymbosum* L. (survey carried out by “Association of Blueberry Producers of Mesopotamia Argentina”, APAMA for its acronym in Spanish, in 2017), representing approximately 39% of the national cultivated area and one of the main exports to Europe and the United States (statistical information Argentinean Blueberry Committee, 2014). This crop requires insect-mediated pollination for optimal productive yields^25,26^, in which the deposition of compatible pollen in the stigmas during flowering produces an increase in both fruiting and size of the berries^27^. To achieve a high pollination rate of this crop, growers promote the presence or rent hives of *A. mellifera* and are willing to provide their fields with high densities of bees^28^. However, the existence of different management practices produces variable scenarios that range from the non-use of bees to having several hives within the productive properties. The majority of the producers who rely on managed beehives to sustain the pollination of their crop use a stocking rate that oscillates between 6 and 12 hives per ha, hoping that they are sufficient for adequate pollination^25,29^.

Within the context described above, the objective of our work was to compare the frequency of visits, and the quantity (fruit-set) and quality (weight and firmness) of fruits produced in plots of blueberry with precision- versus conventional-pollination management practices. In this way, we intend to lay a groundwork for the implementation of good pollination practices using *Apis mellifera* as the pollinator by identifying the following critical points: 1) choice of high quality bee hives to be used, 2) preparation of the hives to enter the field before flowering, 3) hive location and distribution within the field, 4) bee nutrition and management within the field, and 5) bee health such that no pesticides are sprayed during the pollination service period. We hypothesized that plots of *V. corinbosum* managed under the precision pollination service would show higher bee visitation frequencies to their flowers, resulting in an increased proportion and quality of the fruits formed than plots managed under conventional pollination.

## Results

### Honey bee visitation frequency

**Figure 1:**
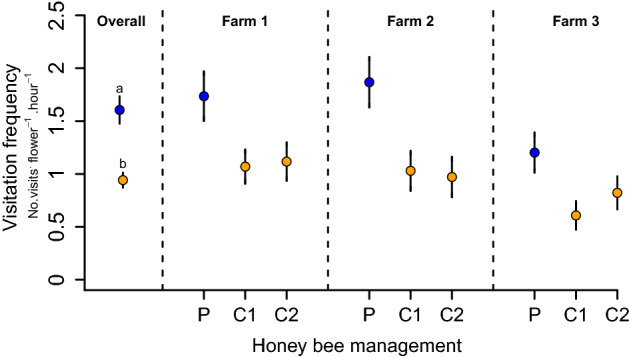
Honey bee visitation frequency. Overall, precision (P) honey bee management fields had higher visitation frequencies to blueberry flowers than conventional (C) honey bee managed fields. C1 and C2 denote each of the two control plots sampled per farm. Points represent mean values, while bars show 1 standard error (SE). *Letters indicate statistical differences between treatments*.

Honey bees were by far the dominant floral visitor to blueberry flowers, accounting for more than 99% of all visits. We observed 1050 honey bee visits to blueberry flowers after almost 50 h of observation. Fields under precision honey bee management had 70% more visitation frequency to flowers than conventional honey bee management (in *log* scale, *β* = 0.53, *SE* = 0.06, *z* = 8.59, *P* < 0.001). Fields under precision and conventional honey bee management showed, on average (± SE), 1.60 ± 0.13 and 0.94 ± 0.07 visits * flower-1 * hour-1, respectively (Fig. 1).

### Fruit set

**Figure 2:**
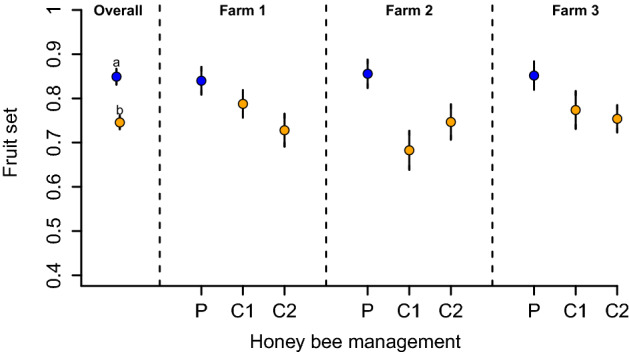
Fruit set. Overall, precision (P) honey bee management fields had higher fruit set than conventional (C) honey bee managed fields. C1 and C2 denote each of the two control plots sampled per farm. Points represent mean fruit set values, while bars show 1 standard error (SE). *Letters indicate statistical differences between treatments*.

Increases in visitation frequency translated into increases of the likelihood of a flower to turn into a fruit. Fields under precision honey bee management produced 13% more fruit set than conventional honey bee management fields (in *logit* scale, *β* = 0.73, *SE* = 0.18, *z* = 3.99, *P* < 0.001). On average, 84% of the flowers set a fruit when precision honey bee management was applied, while fields under conventional management showed 74% of the flowers setting a fruit (Fig. 2).

### Fruit weight

**Figure 3:**
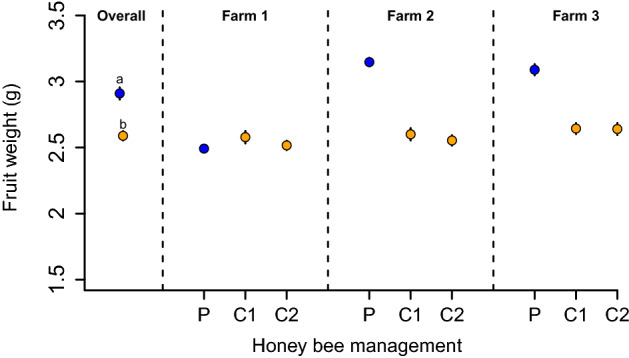
Fruit weight. Overall, precision (P) honey bee management fields had heavier fruits than conventional (C) honey bee managed fields. C1 and C2 denote each of the two control plots sampled per farm. Points represent fruit weight mean values, while bars show 1 standard error (SE). *Letters indicate statistical differences between treatments*.

Following the same trend, fields under precision honey bee management produced fruits 12% heavier than conventional honey bee management fields (*β* = 0.32, *SE* = 0.12, *t* = 2.62, *p* = 0.04). Fruits from fields under precision honey bee managements weighed, on average (± SE), 2.90 ± 0.04 g, while fruits from fields under conventional management weighed 2.58 ± 0.04 g (Fig. 3).

### Fruit firmness

**Figure 4:**
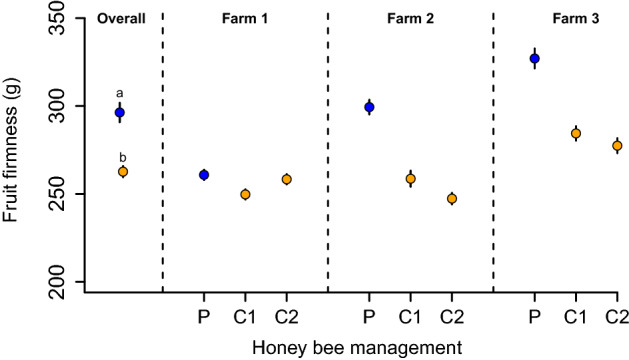
Fruit firmness. Overall, precision (P) honey bee management fields had stronger fruits than conventional (C) honey bee managed fields. C1 and C2 denote each of the two control plots sampled per farm. Points represent mean values, while bars show 1 standard error (SE). *Letters indicate statistical differences between treatments*.

Finally, fields under precision honey bee management produced fruits with 12% higher firmness than conventional honey bee management fields (*β* = 36.10, *SE* = 9.38, *t* = 3.84, *p* = 0.01). Fruits from fields under precision honey bee management showed a firmness, on average (± SE), of 296.62 ± 5.30 g, while fruits from fields under conventional management were 260.53 ± 5.07 g (Fig. 4).

## Discussion

Within the *Vaccinium* genus several species have been domesticated for productive purposes, among which *V. corymbosum* is intensively cultivated to produce quality and bulk fruit in Argentina for export and domestic market purposes (APAMA report 2015). In view of the high dependence of this species on entomophilic pollination, the incorporation of precision honey bee management to maximize crop production is highly needed.

Our work evaluated different relevant aspects of blueberry production from the perspective of planning and managing of the pollination service using *Apis mellifera* hives. This study highlights the effect of precision handling of hives used for such services on the productivity of commercial fields of blueberries.

*Vaccinium corymbosum* var. “Emerald” is highly dependent on entomophilous pollination to obtain optimal production and quality fruit, and even with the conventional pollination service there would be a pollen limitation within productive fields^1^, which is possible to optimize through a precision management of the pollination service as we showed here. The results obtained in our work demonstrate that the correct selection and distribution of bee-hives managed around the focal plots to which the pollination service will be performed is essential, as we found that fields under precision honey bee management had a 70% higher visitation frequency to flowers than conventional honey bee management fields. In blueberry fields in Entre Ríos the pollination season usually begins at the end of winter, and thus bee colonies are small at that time. It is known that performance of small colonies is proportionally lower than large colonies. A healthy average colony may allocate 50–60% of its workers to foraging compared to 35–40% in a small colony^30^. This means that a colony of 40,000 bees will have more foragers than two colonies of 20,000 each. Indeed, colony strength was found to be a strong predictor of pollination value in cranberries^31^. Thus, it is important that the hives to be used for pollination remain healthy with enough bees to pollinate the crop^30,32^, as we did here under precision pollination.

The increase in the frequency of visits by 70% in fields under precision honey bee management translated into 13% more fruit set than conventional honey bee management fields. The positive influence of *A. mellifera* on crops of commercial importance has been widely reported^33–35^, therefore, our results reinforce this knowledge.

In relation to the quality of the harvested fruits for each treatment, the firmness of the fruit and weight showed differences between the treatments, with 12% greater firmness and weight in pollinated berries under precision honey bee management. Differences in the weight and the firmness of the berries were reported in blueberry, although these differences occurred between self-pollination treatments versus different entomophilous pollination treatments^36,37^.

The weight of the fruits and firmness of the pulp is one of the main characteristics for acceptance of the export of the fruit, according to the Quality Protocol for fresh blueberries proposed by the Ministry of Agribusiness in 2015 (Resolution SAGyP N^o^. 201/2007). For this reason, precision honey bee management can not only increase visitation to flowers and fruit production but also fruit quality and post-harvest fruit shelf life. The increase in the firmness of these fruits allows an increase in the useful shelf life of the fruits, better supporting their transport and extending the time that the fruit can be commercialized.

Several studies have recommended different hive densities of *A. mellifera*/ha to provide adequate pollination services in blueberry, which is around ~ 8 hives/ha^32^. Even so, this study is the first to show that an increase in the fruit set of blueberry plants can be attained through differential management techniques with the same number of hives per ha (here we used 10 hives/ha following the local procedures). However, further experiments should be performed to evaluate indicators of the quality of the pollination service (e.g., size of the hive, number of bees, number of visits per unit of time). In view of the results presented in our work, the urgent adoption of precision handling should occur when performing pollination services with bee hives. These practices not only increase the survival of the bees used, but also increase the direct and net economic benefits^38^, and should be part of the agricultural business models. In the case of intensive blueberry production, the benefits of good pollination practices are obvious, evidenced by the fact that it generates an increase, on average, in fruit formation by 13% plus and 12% heavier fruits.

Although there is a lack of knowledge about the processes that explain how changes in visitation frequency to flowers could modify the firmness of blueberry fruit, several authors quote that cell wall calcium levels are determinant in fruit firmness^39,40^. As a hypothesis in this regard, we believe that the effect of the pollinator visit rate on the weight of the fruit (see Fig. 3) could be generating a higher water content within the fruits and thus increasing their firmness. In addition, although all plots sampled had been fertilized (see M&M), soil condition may differ between farms, and between plots within each farm, and thus this variable could explain some of the variation observed in the fruit quality.

Even so, taking into account the average yield values ​​of var. Emerald (10 T/ha.) provided in the market report presented by APAMA in 2015 (https://www.apama.com.ar/index.php), it could be an increase in fruit production of up to 2.656 T/ha, in the case of this variety. If we analyze the earnings resulting from a precision pollination service using *A. mellifera* hives, for the 260 hectares of var. Emerald in the Argentine North East, the increase in profitability per ha would be around 18,592 U$A/ha with a general benefit for the sector of 4,833,920 U$A (considering an average price of 7 U$A/kg of fruit for export). Interestingly, those differential earnings could be obtained with solely an improvement in the pollination service for this variety. From the results obtained in this work and for the reasons detailed above, we encourage blueberry growers and beekeepers to include the practices described here under precision pollination to increase crop production and bee health.

## Methods

### Experimental design

Within the new varieties obtained in the market the variety "Emerald" is a good model for pollination service management. In Argentina the plant is vigorous and very productive, and can produce fruit in autumn without reducing the spring production through the long flowering period^41^. As of 2020, around 260 hectares are planted with this blueberry variety in Entre Ríos province.

Field work was conducted during the 2017 flowering period (from July 17th to August 23rd, 2017) on 9 blueberry plots (*Vaccinium corymbosum* var. Emerald) located in the region of Concordia (Entre Rios, Argentina). Three plots were pollinated under “precision (precision) honey bee management”, while 6 other plots were pollinated under conventional (conventional) honey bee management (two conventional plots per precision plot). We decided to double the number of control plots per farm (conventional bee management) in order to reflect the variability that these agroecosystems present in the study region.

**Figure 5:**
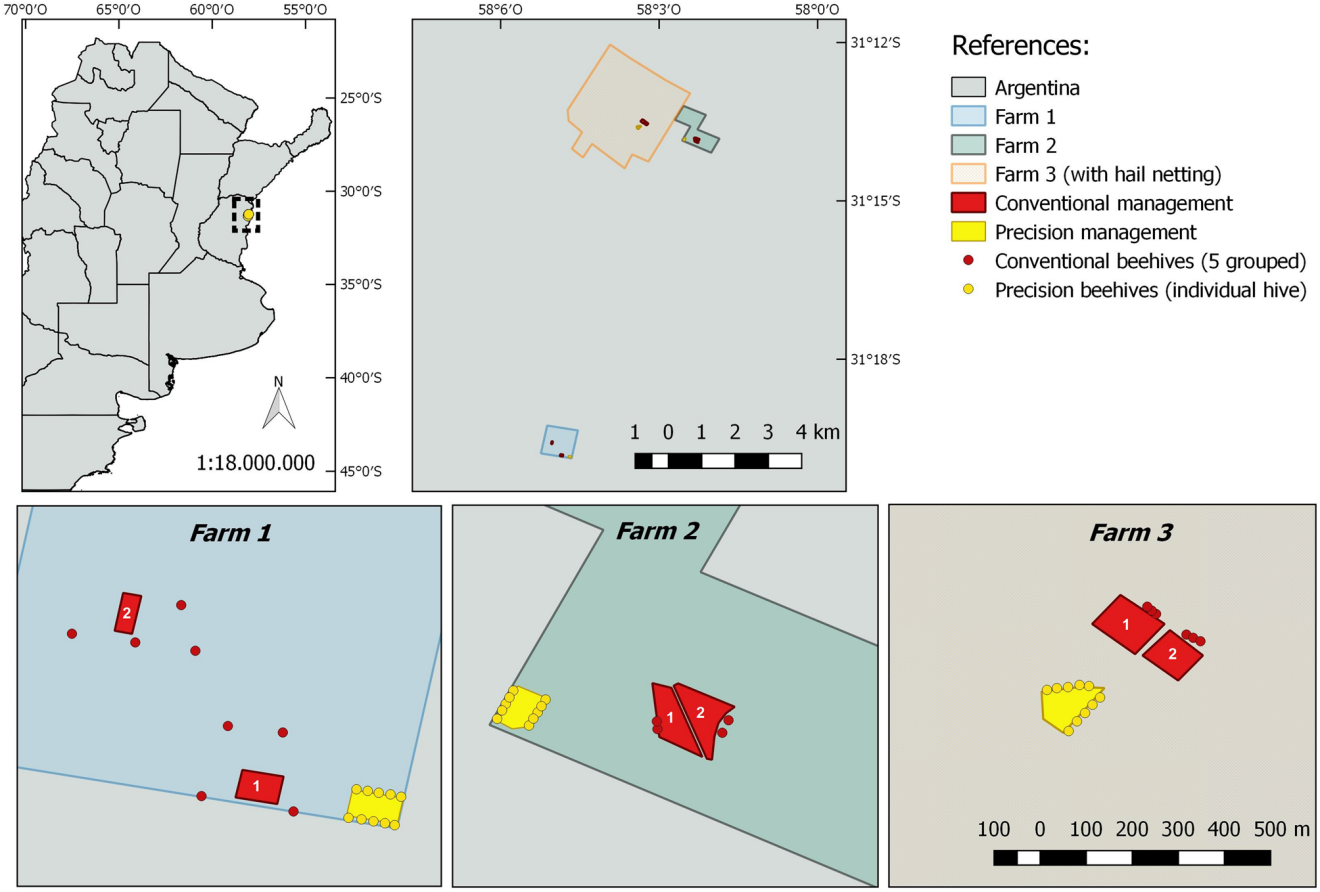
Map of the plots of blueberries studied. The fields with conventional and precision management of bees are detailed in each farm, and the location of the hives used in each particular case. One Precision (yellow) and two Conventional (the numbers stand out C1 and C2, in red) plots were sampled per farm.

Each plot comprised a 1-ha area, planted with the Emerald variety. The plants were all the same age and subjected to the same agricultural management in respect to fertilization, pruning, water, etc. Six of the nine plots (2 precision plots and the corresponding 4 conventional ones) were open air, while 3 plots (one precision and two conventional) were covered with mesh. This mesh is used to prevent hail and wind damage. All the plots were pollinated by honey bees (*Apis mellifera* L.) at a density of 10 hives per cultivated ha (Fig. 5).

#### Precision honey bee management

One month before the blooming season, we carefully selected 30 beehives based on: (a) number of combs covered with adult bees, (b) number of combs with brood, and (c) the presence of an active egg-laying queen, to be prepared for the pollination service. The selected stock of beehives to be used in the precision plots came from the same stock of bees to be used in the control plots, and thus, we could expect that any differences regarding to bees’ performance in the field would be related to the different management (precision) and not due to differences regarding the stocks of bees, neither the ecotypes nor the beekeeping management previous to ours. During the month prior to the pollination service, the selected beehives were prepared for the blooming period by being fed with a dietary supplement (nutritional technology exclusively licensed by Beeflow S.A. and Beeflow Corporation) to boost their immune systems and strength. When blooming reached 5–10% in the precision plots, the beehives were placed so as to surround each plot and managed individually. Beehives were checked during the blooming period and, if it was necessary, they were also fed. We also coordinated pesticide spray applications with growers to avoid bee losses during the pollination service. When 95% of the female flowers were senescent the hives were retired from the field and returned to their original apiary.

#### Conventional bee management

This group of beehives represented the conventional management performed by beekeepers in Concordia Region, which consists of beehives placed in big groups, between 5 to 50, and located either next to or up to hundreds of meters from the field, with high variation in terms of hive quality (see above), and with infrequent servicing. This management style is similar to those performed by many beekeepers around the globe.

In summary, the main differences between the management of bee hives for pollination services in blueberry are: *Start of the incentive diet*, the hives for precision management began to be fed before the blooming period with a dietary supplement (sugar syrup 1: 1) to encourage the collection of pollen; *Protein feeding*, the hives for precision handling were fed during the entire flowering period of the blueberry with a diet especially rich in protein and essential amino acids (i.e.,: alpha arginine); *Spatial arrangement*, strategic and homogeneous location of the hives individually in contrast to distribution in medium groups (5 hives) to large (30 hives) located in strategic places according to the production advisor; *Management against agrochemical applications on the plot*, Preventive management for pesticides, fungicides and fertilizers applied by means of aqueous suspension, in this case through an agricultural spraying turbine for a tractor, was carried out only in the hives for precision pollination.

### Data collection

The visitation rate to flowers was estimated simultaneously in precision and conventional plots, and at different times of the day. To do this, pollinator censuses were performed by recording the number of flowers visited by pollinators for a period of 5 min (i.e., no. visits ^**.**^ flower^−1 .^ 5 min^−1^). Each census involved a different randomly selected group of flowers (between 10 and 30 floral units per census), within a randomized location within the plantation. These visits censuses were carried out in all the lots studied on the same day between 10 am and 6 pm, periodically changing the visiting hours to each lot to cover the entire hourly range throughout the 15 sampling dates distributed in between June 17 and August 23, 2017. Census of visits to flower were carried out on days with a favorable climate for foraging bees, sunny to partially sunny days at times where the temperature it is suitable for bee activity (> 10° C). In total, we performed 70 pollinator censuses per plot distributed in 15 days, totaling 52.5 h of observation (i.e. 5.8 hs per plot × 9 plots).

In each plot, 5 plants were tagged (i.e., 5 plants per plot * 9 plots, 45 plants in total), and within each plant 3 branches were randomly selected and tagged with paper tape (a total of 225 experimental branches). The number of open flowers between the tag and the end of the branch were counted in order to determine the number of flowers that produced a fruit (i.e., fruit set). This experimental design allowed the estimation of variation in flowers within and among plants and plantations, subjected to the different pollination practices.

When the time of the harvest arrived, the number of fruits (corresponding to each tagged branch) developed from each plant was counted in relation to the total number of flowers originally present on the branch to estimate fruit-set. Then, the quality of all tagged fruits harvested was evaluated by quantifying individual weight and firmness. A total of ~ 6700 flowers and ~ 5200 fruits were analyzed.

To estimate the firmness of the fruits we used the "compression force," which is a widely used indicator, using a texture analyzer TA.XT Plus (Stable Micro Systems Ltd., United Kingdom) equipped with a 5 kg load cell and a 75 mm cylinder aluminum probe. It is defined as the force necessary to deform the fruit by 10% and is used as a quantifiable measure equivalent to the force produced when a fruit is pressed between the thumb and the index finger. Finally, to estimate individual fruit weight we used an electronic scale.

### Statistical analysis

We analyzed the effect of honey bee management practice (conventional vs. precision) on: (i) visitation frequency to blueberry flowers, (ii) fruit set, (ii) fruit weight, and (iii) fruit firmness, using general and generalized mixed-effects models. Data analysis was carried out using the *lmer* function from the “*lme4*” package^42,43^ of R software (version 2.15.1), applying separate analyses and distributions for each response variable (e.g. visits frequency, fruit set, and firmness, see below).

For (i) visitation frequency (i.e., bee visits) as the response variable the model assumed a Poisson error distribution with a *log-link* function. We included the number of flowers observed in each census as an offset (i.e., a fixed predictor known in advance to influence insect visitation)^44^. For (ii) fruit set (i.e., proportion of flowers setting fruit) as response variable the model assumed a Binomial error distribution with a *logit-link* function. And finally, for (iii) fruit weight and (iii) fruit firmness as response variables the models assumed a Gaussian error distribution. For all models, honey bee management practice (conventional vs precision) was included as a fixed effect, each “plant” was nested within “field”, and each “field” was nested within “farm” as a random effect, following the hierarchical design of our experimental design (i.e. 3 farms, 3 fields within each farm, and 5 plants within each field).

## Data Availability

We emphasize that the botanical species used (*Vaccinium corymbosum var. Emerald*) is exotic for our country and is reproduced for productive use in commercial nurseries, so our study does not represent any problem for the environment and complies with the local regulations of the regulatory entity "Naturals Resources of the Entre Rios province "in this regard. In addition, we have the pertinent permits from the companies (permits granted by Agroberries S.A. and Blueberry S.A.) where data was collected on the blueberry plants.
